# Formation of Mixed-Ligand Complexes of Pd^2+^ with Nucleoside 5'-Monophosphates and Some Metal-Ion-Binding Nucleoside Surrogates

**DOI:** 10.3390/molecules191016976

**Published:** 2014-10-22

**Authors:** Oleg Golubev, Tuomas Lönnberg, Harri Lönnberg

**Affiliations:** Department of Chemistry, University of Turku, FIN-20014 Turku, Finland; E-Mails: oleg.golubev@utu.fi (O.G.); tuanlo@utu.fi (T.L.)

**Keywords:** Pd^2+^ complexes, nucleosides, NMR, mixed-ligand complexes

## Abstract

Formation of mixed-ligand Pd^2+^ complexes between canonical nucleoside 5'-monophosphates and five metal-ion-binding nucleoside analogs has been studied by ^1^H-NMR spectroscopy to test the ability of these nucleoside surrogates to discriminate between unmodified nucleobases by Pd^2+^-mediated base pairing. The nucleoside analogs studied included 2,6-bis(3,5-dimethylpyrazol-1-yl)-, 2,6-bis(1-methylhydrazinyl)- and 6-(3,5-dimethylpyrazol-1-yl)-substituted 9-(β-d-ribofuranosyl)purines **1**–**3**, and 2,4-bis(3,5-dimethylpyrazol-1-yl)- and 2,4-bis(1-methylhydrazinyl)-substituted 5-(β-d-ribofuranosyl)-pyrimidines **4**–**5**. Among these, the purine derivatives **1**-**3** bound Pd^2+^ much more tightly than the pyrimidine derivatives **4**, **5** despite apparently similar structures of the potential coordination sites. Compounds **1** and **2** formed markedly stable mixed-ligand Pd^2+^ complexes with UMP and GMP, UMP binding favored by **1** and GMP by **2**. With **3**, formation of mixed-ligand complexes was retarded by binding of two molecules of **3** to Pd^2+^.

## 1. Introduction

It has been well established that linear-coordinating Hg^2+^ and Ag^+^ ions may stabilize TT and CC mismatches within oligonucleotide duplexes while square-planar-coordinating Cu^2+^ ion is able to bridge various modified metal-ion-binding bases on opposite strands [1,2,3]. Much less is known about discrimination between canonical nucleobases by oligonucleotide probes incorporating metal-ion-binding surrogate bases. Cu^2+^ and Zn^2+^ ions have been shown to enhance hybridization of 2'-*O*-methyl oligoribonucleotides containing a 2,6-bis(3,5-dimethylpyrazol-1-yl)purine base with complementary unmodified 2'-*O*-methyl oligoribonucleotides [4]. The magnitude of duplex stabilization does not, however, depend only on the identity of the opposite base, but also on the flanking sequences.

**Figure 1:**
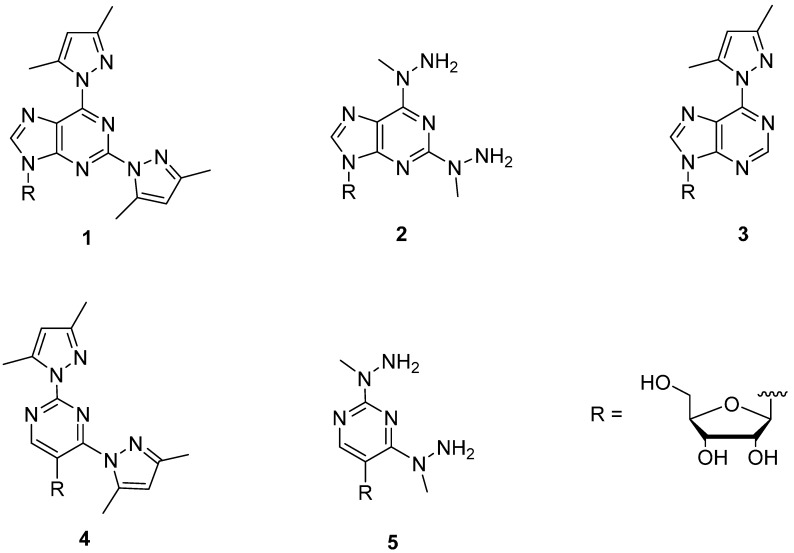
Structures of the metal-ion-binding nucleosides **1**–**5** used in the present study.

**Figure 2:**
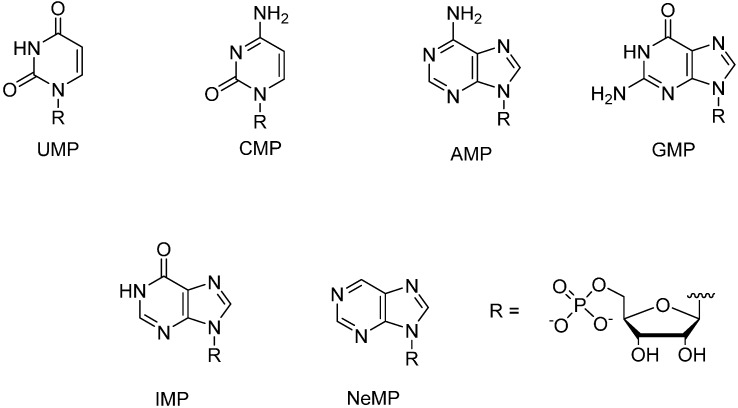
Structures of nucleoside 5'-monophosphates used in the present study.

Square-planar-coordinating Pd^2+^ ion is known to exhibit exceptionally high affinity to nucleic acid bases [5,6,7]. Accordingly, Pd^2+^ complexes are interesting candidates for base moiety discrimination, although so far no convincing examples of Pd^2+^-mediated base-pairing at oligonucleotide level are available. Only some indications of recognition of thymine within an oligodeoxyribonucleotide by 2,6-bis(3,5-dimethylpyrazol-1-yl)purine in the presence of Pd^2+^ have been observed [8]. We have previously tried to evaluate the formation of mixed-ligand Pd^2+^ complexes between some metal ion binding nucleoside analogs and pyrimidine nucleosides [9]. Owing to severe solubility problems, the results obtained remain scanty. To learn more about the discrimination power of Pd^2+^ complexes, we now report on NMR studies concerning formation of mixed-ligand Pd^2+^ complexes between metal-ion-binding nucleosides **1**–**5** (Figure 1) and six nucleoside 5'-monophosphates (NMPs, Figure 2).

## 2. Results and Discussion

### 2.1. Compounds Employed

The preparation of metal-ion-binding nucleosides **1**–**5** has been described previously [9,10]. Among the NMPs employed, UMP, CMP, AMP, GMP and IMP were commercial products and the preparation of the 5'-monophosphate of nebularine (NeMP), *i.e.*, unsubstituted 9-(β-d-ribofuranosyl) purine, has been described previously [11].

### 2.2. Pd^2+^ Complexes of Modified Nucleosides *(**1**–**5**)*

Interaction of the modified nucleosides **1**–**5** with Pd^2+^ was studied first. For this purpose, K_2_PdCl_4_ was added portionwise into a 5.0 mmol·L^−1^ solution of the nucleoside in phosphate buffered D_2_O (0.12 mol·L^−1^, pD 7.6, 25 °C) keeping the nucleoside concentration constant. After each addition a ^1^H-NMR spectrum was recorded. Table 1 records the chemical shifts of the signals referring to the Pd^2+^ complexes formed.

Upon addition of K_2_PdCl_4_ to 2,6-bis(3,5-dimethylpyrazol-1-yl)purine riboside (**1**), the intensity of the H8 singlet of **1** at 8.59 ppm gradually decreased and two new pairs of singlets (8.85 and 8.83 and 8.63 and 8.61) appeared (Figure S1 in Supplementary Files). When 0.5 equiv. of K_2_PdCl_4_ had been added, the H8 singlet at 8.59 had almost entirely disappeared and a new singlet at 8.71 appeared. On approaching 1.0 equiv. addition of K_2_PdCl_4_, the two pairs of singlets weakened while the singlet at 8.71 became more intense (Figure S2 in Supplementary Files). Corresponding changes occurred in the anomeric proton region. Formation of the two pairs of singlets at 8.85 and 8.83 and 8.63 and 8.61 was accompanied by the appearance of three anomeric proton doublets at 5.98 (*J* 7.6), 5.89 (*J* 4.4 Hz) and 5.84 (*J* 4.8 Hz), the first one being twice as intense as the latter ones. A doublet at 6.19 (*J* 4.2 Hz), in turn, appeared parallel to the singlet at 8.71. Accordingly, at low concentration of K_2_PdCl_4_, a 2:1 (**1**:Pd) complex is formed and on increasing the concentration of K_2_PdCl_4_, the 1:1 complex predominates.

**Table 1:** Chemical shifts for the aromatic and anomeric protons of the modified nucleosides **1**–**5** and their Pd^2+^ complexes in D_2_O at pD 7.6 (0.12 M phosphate buffer, 25 °C).

Compd.	Aromatic Proton Shifts	Anomeric Proton Shifts
**1**	s 8.59(H8), s 6.19 and 6.11(H4'')	d 6.15 (*J* 5.2)
(**1**)Pd	s 8.71(H8) *^a^*	d 6.19 (*J* 4.2)
(**1**)_2_Pd	s 8.85 and 8.83 and 8.63 and 8.61(H8) *^b^*	d5.98(*J* 7.6), d 5.89 (*J* 4.4), d 5.84(*J* 4.8)
**2**	s 7.86(H8)	d 5.84 (*J* 5.3)
(**2**)Pd	s 8.06(H8)	d 5.86 *^c^*
**3**	s 8.81(H2), s 8.63(H8), s 6.23(H4'')	d 6.16 (*J* 5.6)
(**3**)Pd	s 8.92(H2/8), s 8.70(H2/8), s 6.40(H4'')	d 6.19 (*J* 3.8)
	s 8.84(H2/8), s 8.42(H2/8), s 6.38(H4'')	d 6.19 (*J* 3.8)
**4**	s 9.03(H6), s 6.12 and 6.07(H4'')	d 5.06 (*J* 5.4)
(**4**)Pd	*d*	*e*
**5**	s 7.92(H6)	s 5.60

*^a^* H4'' signals at 6.20–6.45 overlap with the corresponding signals of (**1**)_2_Pd. *^b^* Several H4'' signals at 6.20–6.64. *^c^* Overlaps with H1' of **2**. *^d^* The disappearance of H6 singlet of uncomplexed **4** was accompanied by appearance of 6 new singlets at 9.28, 9.22, 9.20, 8.93, 8.63 and 8.60. *^e^* The disappearance of the H1' doublet of **4** was accompanied by appearance of 3 new signals at 5.73, 5.39 and 5.30.

The 1:1 complex, exhibiting only one set of ^1^H-NMR signals, most likely is a (**1**)PdCl^+^ complex, the metal ion being coordinated to N1 of the purine base and N2 atoms of the pyrazolyl substituents. When the concentration of **1** is high compared to that of K_2_PdCl_4_, the chlorido ligand is replaced with another molecule of **1** which undergoes either N1 or N7 binding. N7 binding appears more likely, since this site is sterically less hindered than the N1 site flanked by the 3,5-dimethylpyrazol-1-yl groups, and since a reasonably large (0.26 and 0.24 ppm) downfield shift of the H8 signal is observed [7,12,13,14,15]. The H8 resonances of both modified purine bases engaged in the complex appear as two singlets, most likely due to the fact that two mutual orientations of the ligands are possible: the sugar moieties may be situated on the same or opposite sides of the plane of Pd^2+^ and the purine bases.

6-(3,5-Dimethylpyrazol-1-yl)purine riboside (**3**) bound Pd^2+^ much more weakly. Only half of **3** was complexed at an equimolar 5 mmol·L^−1^ concentration (Figure S3 in Supplementary Files). Two complexes were formed in parallel, evidently due to almost as efficient binding to N1 and N7 in addition to binding to the pyrazolyl N2 atom. The markedly lower affinity compared to **1** lends substantial additional evidence for the assumption that both pyrazolyl groups of **1** participate in binding of Pd^2+^.

Another important observation is that replacement of aromatic 3,5-dimethylpyrazol-1-yl groups with aliphatic 1-methylhydrazinyl groups markedly weakens the binding of Pd^2+^. Only half of 2,6-bis(1-methylhydrazinyl)purine riboside (**2**) was engaged in complex formation at 5.0 mmol·L^−1^ concentration of K_2_PdCl_4_ and **2**, although a tridentate coordination, as with **1**, apparently is possible (Figure S4 in Supplementary Files). However, binding to the terminal amino groups of the hydrazinyl substituents is evidently impeded by the fact that the lone electron pair of the nitrogen atoms participates in the π-electron resonance of the purine ring, which lowers the electron density at the potential donor atoms. With **1** the situation is different, since the lone electron pair of the N2 atoms of the pyrazolyl substituents is not delocalized but the N2 atoms are pyridine type nitrogens. Additionally, hydrogen bonding of the NH_2_ group to N1 gives an expectedly moderately stable five membered structure, which may still retard the complexing ability of **2**. A marked broadening of the signals took place at high concentrations of K_2_PdCl_4_ and unidentified broad signals appeared, which may well refer to formation of polymeric complexes.

**Figure 3:**
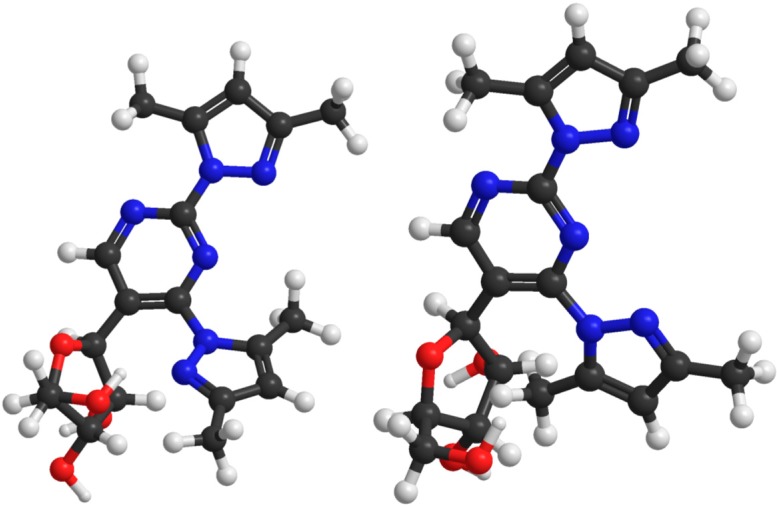
Semi-empirical (PM6) minimized structure for 2,4-bis(3,5-dimethylpyrazol-1-yl) -5-(β-d-ribofuranosyl)pyrimidine (**4**, **left**) and the structure allowing tridentate binding to the N2 atoms of the 3,5-dimethylpyrazolyl groups and the intervening N3 of the pyrimidine ring (**right**). In the latter structure the steric repulsion is much more pronounced than in the former.

Quite unexpectedly, 2,4-bis(3,5-dimethylpyrazol-1-yl)-5-(β-D-ribofuranosyl)pyrimidine (**4**) also turned out to bind Pd^2+^ very weakly in spite of the fact that the expected binding site, *viz.* the N2 atoms of the two pyrazolyl groups and the intervening N3 of the pyrimidine ring, appears very similar to the binding site in **1**. At 5.0 mmol·L^−1^ concentration of both K_2_PdCl_4_ and **4**, more than 50% of **4** was complexed, but instead of a single clearly recognizable tridentate complex, several species in comparable amounts were formed (Figure S5 in Supplementary Files). Presumably, steric repulsion between the ribosyl group and the 5-methyl substituent of the pyrazolyl group at C4 prevents this group to adopt a coplanar orientation with the pyrimidine and the other pyrazolyl ring required for tridentate binding of Pd^2+^ (Figure 3). In other words, owing to this repulsion, the N2 side of the prazolyl group is turned away from the vicinity of the N3 binding site.

2,4-Bis(1-methylhydrazinyl)-5-(β-d-ribofuranosyl)pyrimidine (**5**) binds Pd^2+^ even more weakly than **4**. In fact, no signals referring to complex formation could be detected upon addition of K_2_PdCl_4_ into a 5 mmol·L^−1^ solution of **5**. As with **2**, involvement of the lone electron pair of the N2 atom of the 1-methylhydrazinyl groups in the π-electron resonance of the heteroaromatic ring makes the hydrazinyl amino groups poor donor atoms, but does not explain why binding of Pd^2+^ to **5** is even weaker than binding to **2**. Tentatively, the presence of the bulky ribofuranosyl group next to one of the hydrazinyl groups still sterically retards the binding of Pd^2+^.

### 2.3. Mixed-Ligand Pd^2+^ Complexes of Modified Nucleosides ***1**–**5*** with Nucleoside 5'-Monophosphates

**Figure 4:**
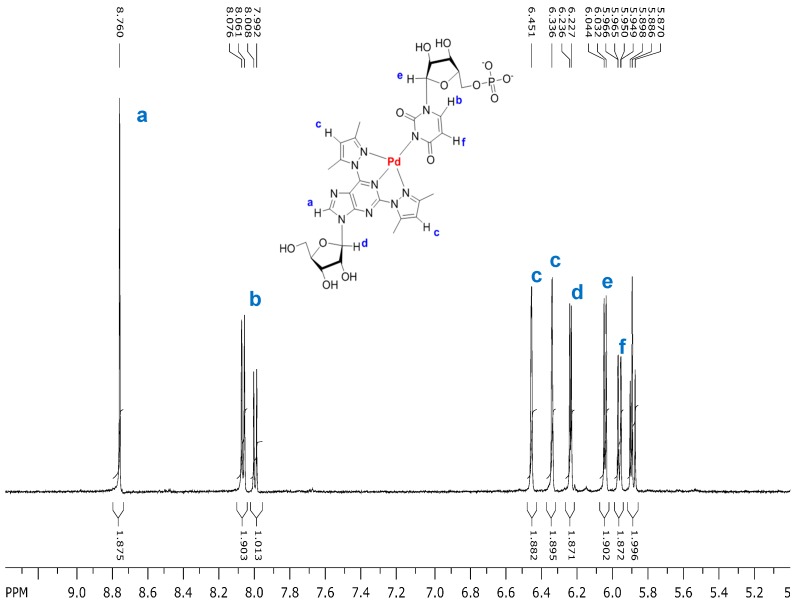
Partial ^1^H-NMR spectrum of a mixture of 2,6-bis(3,5-dimethylpyrazol-1-yl)purine riboside (**1**; 3.4 mmol·L^−1^), K_2_PdCl_4_ (3.4 mmo·L^−1^) and UMP (5.0 mmol·L^−1^) in D_2_O (phosphate buffer 0.12 mol·L^−1^, pD 7.6, 25 °C). The doublet at 8.00 and overlapping doublets at 5.89 refer to uncomplexed UMP.

Since 2,6-bis(3,5-dimethylpyrazol-1-yl)purine riboside (**1**) formed by far the most stable Pd^2+^ complexes among the modified nucleosides studied, the formation of mixed-ligand Pd^2+^ complexes between this nucleoside and various NMPs was then studied. For this purpose, equimolar amounts of **1** and K_2_PdCl_4_ were stepwise added into a 5.0 mmol·L^−1^ solution of NMP in phosphate buffered D_2_O (0.12 mol·L^−1^, pD 7.6, 25 °C), keeping the concentration of NMP constant. Upon addition of **1** and K_2_PdCl_4_ into the solution of UMP, the ^1^H-NMR signals of UMP gradually disappeared and a set a signals referring to a mixed-ligand Pd^2+^ complex of **1** and UMP appeared. Figure 4 shows as an illustrative example the spectrum obtained when the total concentration of **1** and K_2_PdCl_4_ was 3.4 mmol·L^−1^. The chemical shifts of the aromatic and anomeric proton resonances are given in Table 2. No signals referring to the complex (**1**)Pd^2+^ or (**1**)_2_Pd^2+^ appeared. Most likely, deprotonated N3 of UMP occupies the fourth coordination site of Pd^2+^ bound tridentately to **1**. When the total concentration of **1**, K_2_PdCl_4_ and UMP was 4.0, 4.0 and 5.0 mmol·L^−1^, respectively, 78% of UMP was engaged in the mixed ligand complex, the theoretical maximum being 80% (Table 3). These conditions were selected as reference conditions in the present study, since at equimolar 5.0 mmol concentration considerable broadening of NMR signals in some cases occurred, which may be taken as an indication of polymeric complex formation or precipitation.

**Table 2:** Chemical shifts for the aromatic and anomeric protons of the mixed ligand Pd^2+^ complexes of 2,6-bis(3,5-dimethylpyrazol-1-yl)purine riboside (**1**) with nucleoside 5'-monophosphates in D_2_O at pD 7.6 (0.12 M phosphate buffer, 25 °C).

Compd.	Aromatic Proton Shifts	Anomeric Proton Shifts
UMP	d 8.00(H6) *^a^*, d 5.88(H5) *^a^*	d 5.88 *^b^*
(**1**)Pd(UMP)	s 8.76(H8 of **1**), s 6.45 and s 6.33(H4'' of **1**), d 8.07(H6 of UMP) *^c^*, d 5.96(H5 of UMP) *^c^*	d 6.23(*J* 4.6), d 6.04(*J* 5.6)
CMP	d 7.98(H6) *^d^*, d 6.03(H5) *^d^*	d 5.89(*J* 5.2)
(CMP)Pd	d 7.93(H6) *^d^*, d 5.92(H5) *^d^*	d 5.82(*J* 5.5)
(**1**)Pd(CMP)	s 8.59(H8 of **1**), s 6.48 and 6.37(H4'' of **1**), d 7.86(H6 of CMP) *^d^*, d 5.94(H5 of CMP) *^d^*	d 5.83(*J* 3) d 5.83(*J* 3.9), d 5.88 *^e^*
GMP	s 8.09(H8)	d 5.82(*J* 6.1)
(**1**)Pd(GMP)	s 8.59(H8 of **1**), s 6.43 and 6.32 (H4'' of **1**), s 8.11(H8 of GMP)	d 6.14(*J* 5.3), d 5.85(*J* 6.3)
IMP	s 8.46(H8), s 8.12(H2)	d 6.03(*J* 5.8)
(**1**)Pd(IMP)	s 8.75 (H8 of **1**), s 6.40 and 6.29(H4'' of **1**), s 8.66(H8 of IMP), s 8.44(H2 of IMP)	d 6.02 *^e^*, d 6.14(*J* 5.3)
AMP *^f^*	s 8.48(H8), s 8.14(H2)	d 6.02 (*J* 5.9)

*^a^* *J* 8.2 Hz. *^b^* Overlaps with H5. *^c^* *J* 7.8 Hz. *^d^* *J* 7.6 Hz. *^e^* Overlaps with the H1' resonance of the uncomplexed NMP. *^f^* (AMP)Pd and (**1**)Pd(AMP) precipitated.

**Table 3:** Mole fraction of NMPs engaged in a mixed-ligand Pd^2+^ complex with modified nucleosides **1**–**5**, when the total concentration of **1**, K_2_PdCl_4_ and NMP is 4.0, 4.0 and 5.0 mmol·L^−1^, respectively.

NMP	1	2	3	4	5
UMP	0.78	0.41	*d*	*d*	*b*
CMP	≈0.2	0.26	*d*	*b*	*d*
GMP	0.45	0.61	*d*	*b*	*b*
IMP	0.57	*c*	*c*	*c*	*c*
AMP	*a*	*d*	*b*	*b*	*b*
NeMP	*b*	*c*	*c*	*c*	*c*

*^a^* Precipitation occurred. *^b^* No mixed-ligand complex formed. *^c^* Not studied. *^d^* Traces of several species formed in parallel.

Mixed ligand complex formation of **1** with the other NMPs is considerably weaker: 45% of GMP and 57% of IMP was engaged in the mixed ligand complex under the reference conditions ([**1**] = [K_2_PdCl_4_] = 4.0 mmol·L^−1^ and [NMP] = 5.0 mmol·L^−1^) (Figures S6 and S7 in Supplementary Files). The binding site cannot be definitely assigned. The H8 signal of GMP and the H2 and H8 signals of IMP all undergo a modest downfield shift, the shift of H2 of IMP being the largest. This suggests that the binding site is deprotonated N1, since N7 binding to a purine base usually shifts the H8 signal downfield by approximately 0.5 ppm, leaving the H2 shift almost unchanged [7,11,12,13,14]. Now the H2 signal of IMP is shifted more than the H8 signal.

In the case of CMP, formation of binary (CMP)Pd^2+^ complex competed with formation of the mixed-ligand complex. Only around 20% of CMP was engaged in the mixed ligand complex under the reference conditions (Figure S8 in Supplementary Files). Similarly, the interaction with purine riboside 5'-monophosphate turned out to be weak; no assignable mixed ligand complex was formed. The binary and mixed ligand Pd^2+^ complexes of AMP precipitated. Accordingly, only NMPs having a displaceable proton at N1 seem to form reasonably stable mixed ligand Pd^2+^ complexes with **1**, UMP being bound considerably more firmly than IMP or GMP.

Among the other modified nucleosides studied (**2**–**5**), only 2,6-bis(1-methylhydrazinyl)purine riboside (**2**) formed mixed ligand complexes stable enough to be reliably detected (Table 4). The complex with UMP was less stable than the corresponding complex of **1**, consistent with the lower affinity of **3** for Pd^2+^. Only 41% of UMP was engaged in the mixed-ligand complex under the reference conditions ([K_2_PdCl_4_] = [**3**] = 4.0 mmol·L^−1^, [NMP] = 5.0 mmol·L^−1^] (Figure S9 in Supplementary Files). For comparison, the observed 78% engagement of UMP in the mixed ligand complex with **1** was close to the theoretical maximum, 80%. Ternary complexes (**2**)Pd^2+^(CMP) and (**2**)Pd^2+^(GMP) were, in turn, formed even slightly more readily than the corresponding complexes of **1**; 26% of CMP and 61% of GMP were engaged in a mixed ligand complex under the reference conditions (Figures S10 and S11 in Supplementary Files). In fact, GMP was now bound slightly more firmly than UMP. The marked downfield shift of the H8 resonance of GMP (0.66 ppm) suggests N7 coordination. Interaction with AMP appeared rather weak, and several species were formed in parallel. Upon mixing of 6-bis(3,5-dimethylpyrazol-1-yl)purine riboside (**3**) and K_2_PdCl_4_ with UMP, CMP or GMP, so complicated mixtures were formed that assignment of any single mixed-ligand complex was impossible. With AMP, no complexes were formed. As discussed above, the pyrimidine derivatives **4** and **5** did not form stable Pd^2+^ complexes. Expectedly, they did not form an assignable mixed-ligand complex with any of the NMPs studied. The only species that could be assigned referred to binary Pd^2+^ complexes of NMPs.

**Table 4:** Chemical shifts for the aromatic and anomeric protons of the mixed ligand Pd^2+^ complexes of 2,6-bis(1-methylhydrazinyl)purine riboside (**2**) with nucleoside 5'-monophosphates in D_2_O at pD 7.6 (0.12 M phosphate buffer).

Compd.	Aromatic Proton Shifts	Anomeric Proton Shifts
(**2**)Pd(UMP)	s 8.13(H8 of **2**), d 7.81(H6 of UMP) *^b^*, d 5.77(H5 of UMP) *^b^*	m 5.91–5.95 *^a^*
(**2**)Pd(CMP)	s 8.14(H8 of **2**), d 8.11(H6 of CMP) *^c^*, d 6.13(H5 of CMP) *^c^*	m 5.90–5.95 *^a^*
(**2**)Pd(GMP)	s 8.04(H8 of **2**), s 8.75(H8 of GMP)	br s 5.81 *^d^*
(**2**)Pd(AMP)	*e*	*e*

*^a^* The H1' resonances of both ligands overlap. *^b^* *J* 7.7 Hz. *^c^* *J* 7.6 Hz. *^d^* Overlaps with the H1' resonance of GMP. *^e^* Could not be reliably assigned.

## 3. Experimental Section

### 3.1. General Information

The ^1^H-NMR spectra were recorded on Bruker Avance 500- or 400-MHz NMR spectrometers using Me_4_Si as an external standard. The chemical shifts, δ, are given in ppm and the coupling constants, *J*, in Hz. HR-ESI-MS spectra were obtained by a Bruker Daltonics MicrOTOF-Q instrument.

### 3.2. ^1^H-NMR Spectroscopic Studies of the Interaction of K_2_PdCl_4_ with Nucleosides ***1**–**5***

To a solution of nucleoside **1**–**5** (5.0 mmol·L^−1^) in a phosphate buffer in D_2_O (0.12 mol·L^−1^, pH 7.2), K_2_PdCl_4_ was added portionwise keeping the concentration of nucleoside constant. After each addition, a ^1^H-NMR spectrum was recorded at 25 °C and the signals appearing in the region of aromatic and anomeric protons (chemical shift > 5 ppm) were carefully integrated. The species distribution at different concentrations was calculated on the basis of the relative intensities of the resonances of their aromatic and anomeric protons.

### 3.3. ^1^H-NMR Spectroscopic Studies of the Formation of Mixed-Ligand Pd^2+^ Complexes of Nucleosides ***1**–**5*** with NMPs

To a solution of NMP (5.0 mmol·L^−1^) in a phosphate buffer in D_2_O (0.12 mol·L^−1^, pD 7.6), a 1:1 mixture of K_2_PdCl_4_ and one of nucleosides **1**–**5** in the same buffer was portionwise added. The concentration of K_2_PdCl_4_ and the nucleoside (**1**–**5**) was in this manner varied from zero to 5 mmol·L^−1^, while the concentration of NMP was kept constant (5.0 mmol·L^−1^). After each addition, a ^1^H-NMR spectrum was recorded at 25 °C and the signals appearing in the region of aromatic and anomeric protons (chemical shift > 5 ppm) were carefully integrated. The species distribution at different concentrations was calculated on the basis of the relative intensities of the resonances of their aromatic and anomeric protons.

## 4. Conclusions

In spite of the apparent similarity of tridentate coordination sites in purine ribosides **1** and **2**, on the one hand, and in pyrimidine *C*-ribosides **4** and **5**, on the other hand, only the purine derivatives turned out to be able to form stable Pd^2+^ complexes. Evidently the bulky ribosyl group at C5 of the pyrimidine ring sterically prevents the donor atom of the C4-substituent from adopting an orientation allowing tridentate binding. Among the purine derivatives, 2,6-bis(3,5-dimethylpyrazol-1-yl)purine riboside (**1**) forms mixed-ligand Pd^2+^ complexes with NMPs much more readily than its 2,6-bis(1-methylhydrazinyl) counterpart, probably due to participation of the lone electron pair of the terminal amino groups of the latter in the purine π-electron resonance. UMP is recognized most efficient, followed by IMP, GMP and CMP, in this order. Interestingly, interaction with unsubstituted purine riboside 3'-monophosphate is very weak. Mixed-ligand complexes with AMP precipitate. Bidentately coordinating 6-(3,5-dimethylpyrazol-1-yl)purine riboside give a complicated product mixture upon mixing with K_2_PdCl_4_ and any of the canonical NMPs.

## Supplementary Materials

Supplementary materials can be accessed at: http://www.mdpi.com/1420-3049/19/10/16976/s1.

## Supplementary Files

Supplementary File 1
