# Supplementary material for: Formation of Mixed-Ligand Complexes of Pd2+ with Nucleoside 5'-Monophosphates and Some Metal-Ion-Binding Nucleoside Surrogates
Source: Molecules. 2014 Oct 22;19(10):16976–86. doi: 10.3390/molecules191016976 (PMC6271181; doi:10.3390/molecules191016976)

Supplementary Materials

Figure S1. Aromatic and anomeric proton signals for the mixture of 2,6-bis(3,5-dimethylpyrazol-1-yl)-9-β-D-ribofuranosylpurine (**1**, 5.0 mmol·L^{-1}) and K_2PdCl_4 (2.0 mmol·L^{-1}) in in D_2O (phosphate buffer 0.12 mol·L^{-1}, pD 7.6, 25 °C). Notation: Open circles refer to uncomplexed **1** and triangles to a 1:2 complex (Pd:**1**).

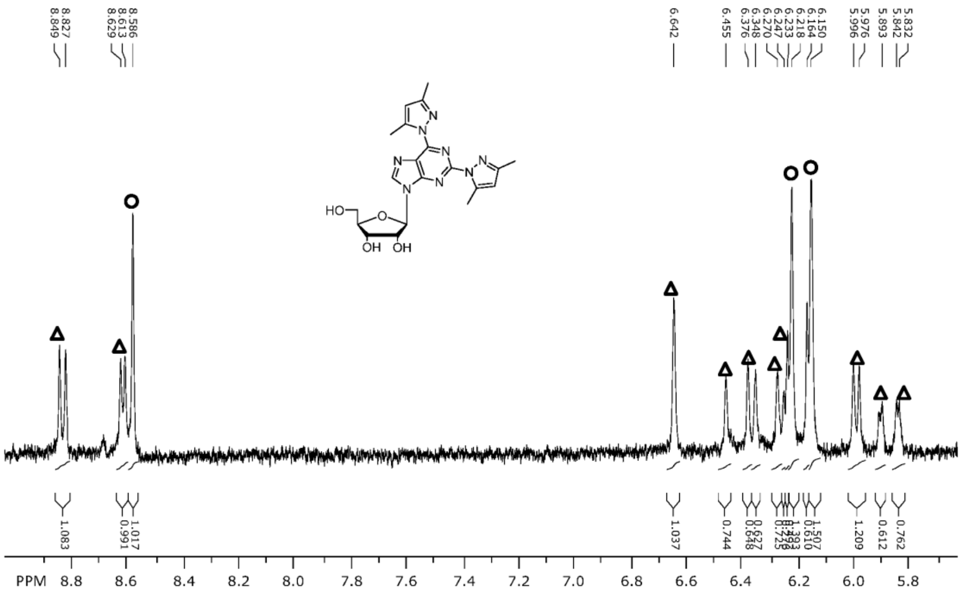

Figure S2. Aromatic and anomeric proton signals for the mixture of 2,6-bis(3,5-dimethylpyrazol-1-yl)-9-β-D-ribofuranosylpurine (**1**, 5.0 mmol·L^{-1}) and K_2PdCl_4 (4.0 mmol·L^{-1}) in in D_2O (phosphate buffer 0.12 mol·L^{-1}, pD 7.6, 25 °C). Notation: Open circles refer to a 1:1 and triangles to a 1:2 complex (Pd:**1**).

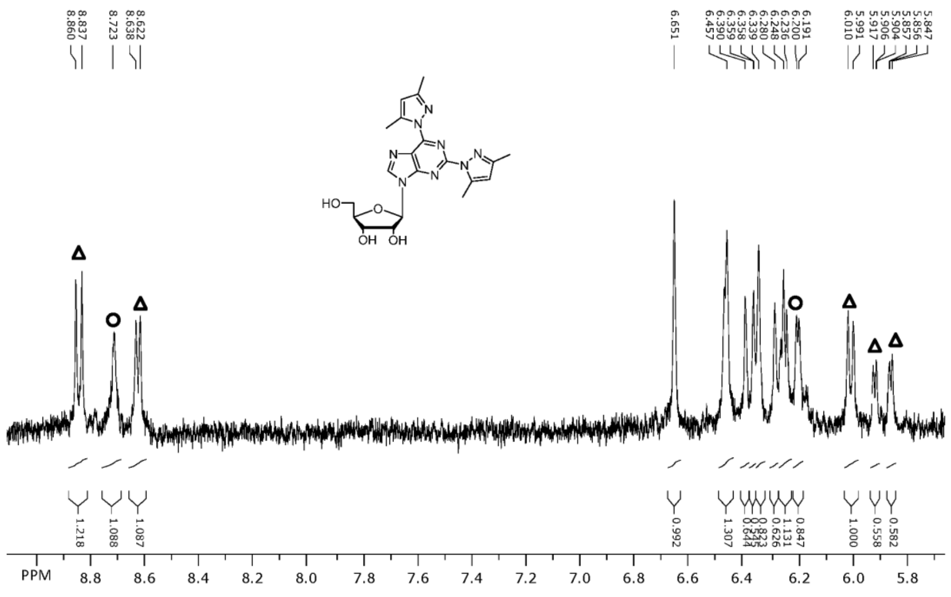

Figure S3. Aromatic and anomeric proton signals for the mixture of 6-(3,5-dimethylpyrazol-1-yl)-9-β-D-ribofuranosylpurine (**3**, 5.0 mmol·L^{-1}) and K_2PdCl_4 (5.0 mmol·L^{-1}) in in D_2O (phosphate buffer 0.12 mol L^{-1}, pD 7.6, 25 °C). Notation: Open circles refer to uncomplexed **3** and open and filled squares to two different 1:1 complexs (Pd:**3**).

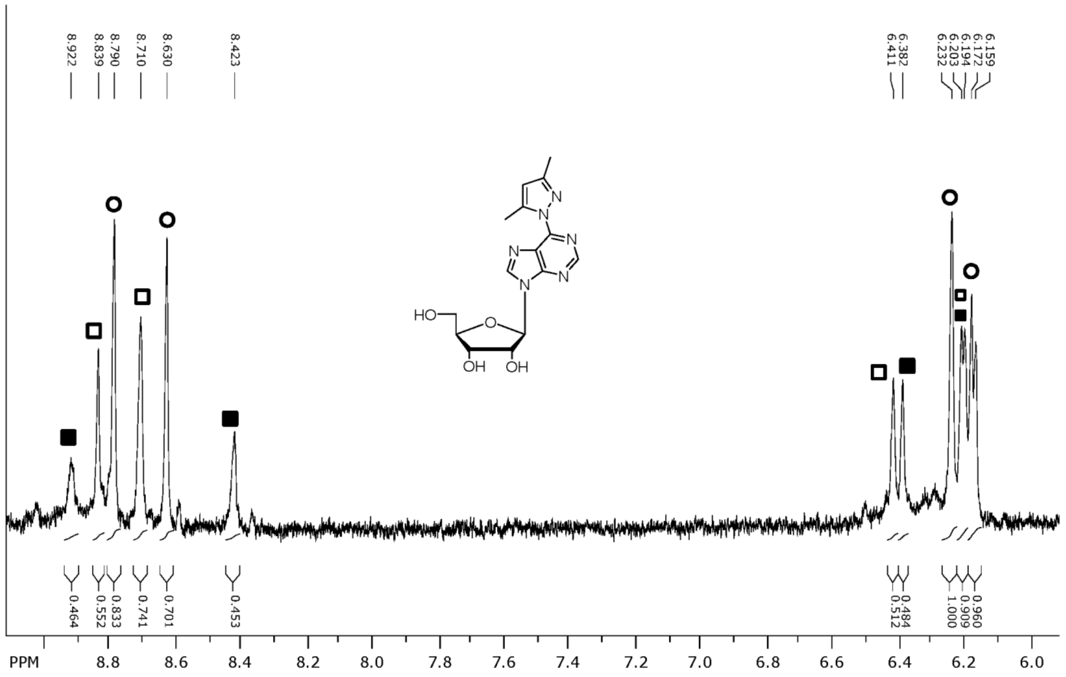

Figure S4. Aromatic and anomeric proton signals for the mixture of 2,6-bis(1-methylhydrazinyl)-9-β-D-ribofuranosylpurine (**2**, 5.0 mmol·L^{-1}) and K_2PdCl_4 (5.0 mmol·L^{-1}) in in D_2O (phosphate buffer 0.12 mol·L^{-1}, pD 7.6, 25 °C). Notation: Open circles refer to uncomplexed **2** and open squares to a 1:1 complexs (Pd:**2**).

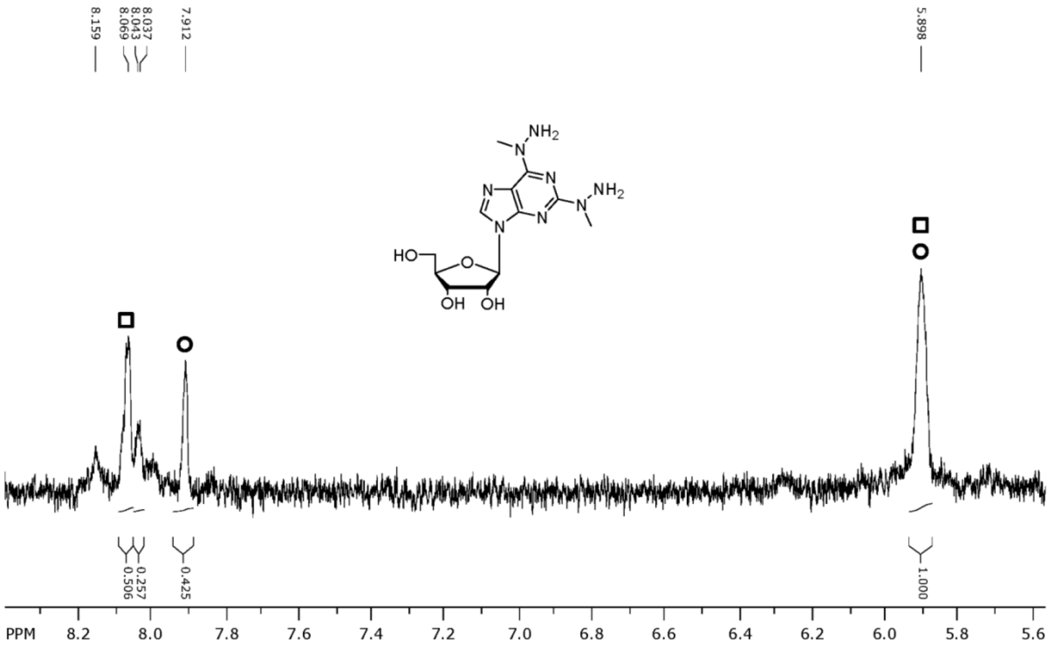

Figure S5. Aromatic and anomeric proton signals for the mixture of 2,4-bis(3,5-dimethylpyrazol-1-yl)-5-(β-D-ribofuranosyl)-pyrimidine (**4**, 5.0 mmol·L^{-1}) and K_2PdCl_4 (5.0 mmol·L^{-1}) in in D_2O (phosphate buffer 0.12 mol·L^{-1}, pD 7.6, 25 °C). Notation: Open circles refer to uncomplexed **4** and the rest of the signals to numerous Pd^{2+} containing species.

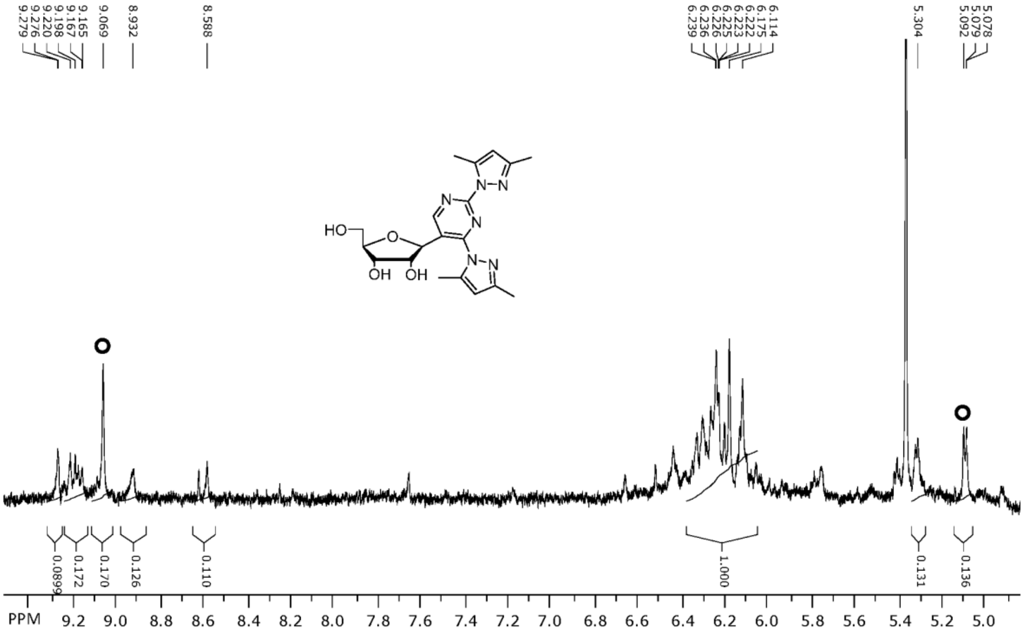

Figure S6. Aromatic and anomeric proton signals for the mixture of GMP (5 mmol·L^{-1}), 2,6-bis(3,5-dimethylpyrazol-1-yl)-9-β-D-ribofuranosylpurine (**1**, 3.0 mmol·L^{-1}) and K_2PdCl_4 (3.0 mmol·L^{-1}) in in D_2O (phosphate buffer 0.12 mol·L^{-1}, pD 7.6, 25 °C). Notation: Open circles refer to uncomplexed GMP and squares to 1:1:1 complexs (**1**:Pd:GMP).

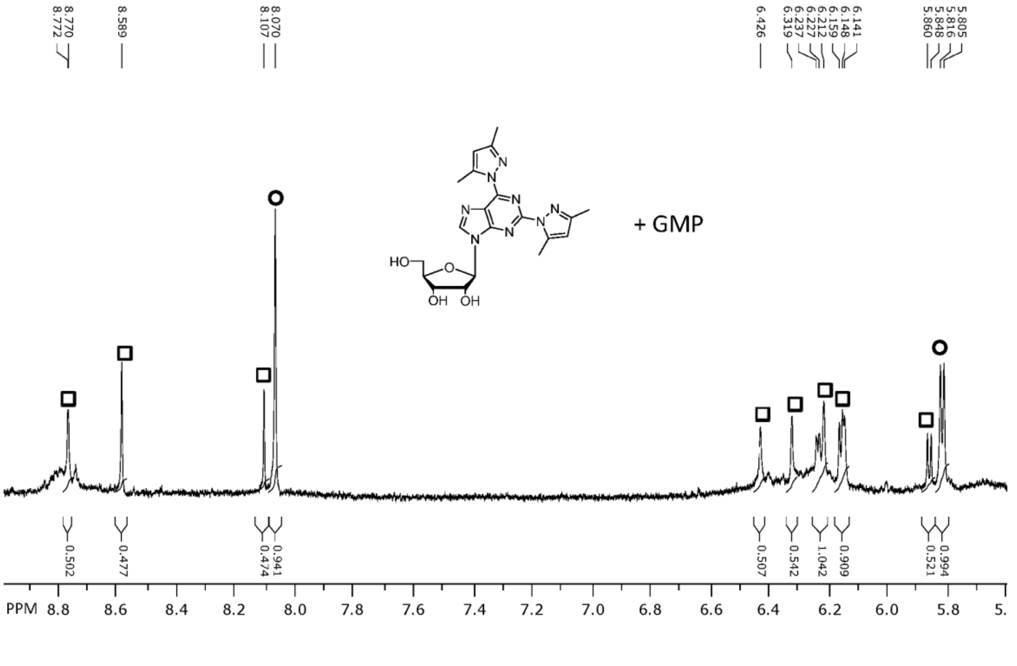

Figure S7. Aromatic and anomeric proton signals for the mixture of IMP (5 mmol·L^{-1}), 2,6-bis(3,5-dimethylpyrazol-1-yl)-9-β-D-ribofuranosylpurine (**1**, 4.0 mmol·L^{-1}) and K_2PdCl_4 (4.0 mmol·L^{-1}) in D_2O (phosphate buffer 0.12 mol·L^{-1}, pD 7.6, 25 °C). Notation: Open circles refer to uncomplexed IMP and squares to 1:1:1 complexs (**1**:Pd:IMP).

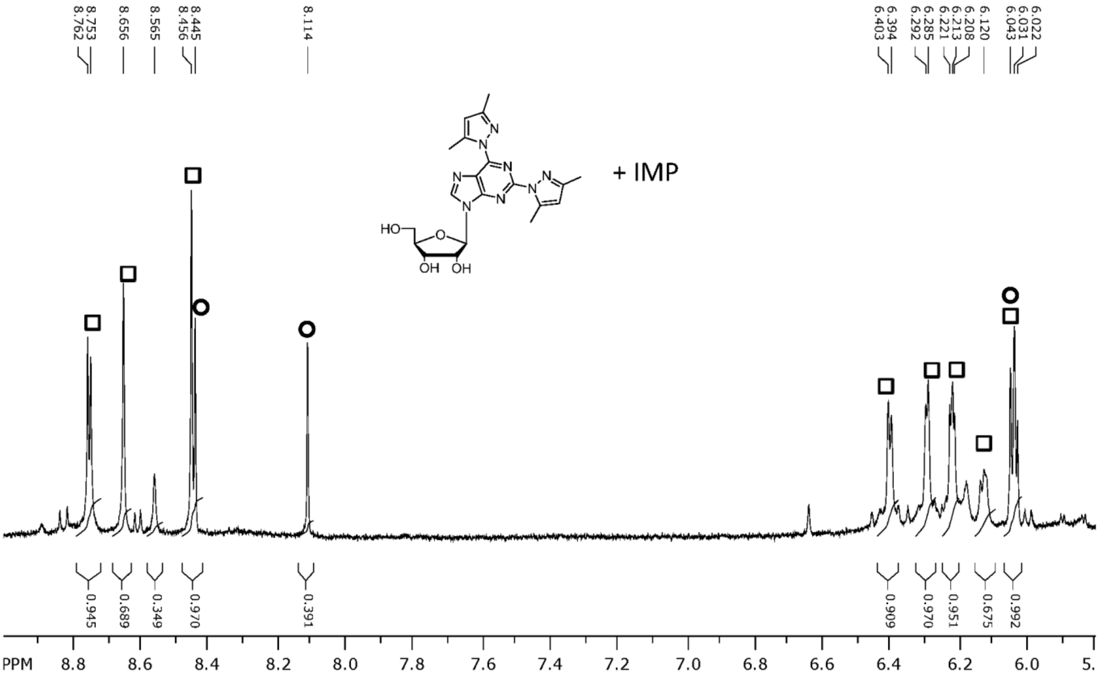

Figure S8. Aromatic and anomeric proton signals for the mixture of CMP (5 mmol·L^{-1}), 2,6-bis(3,5-dimethylpyrazol-1-yl)-9-β-D-ribofuranosylpurine (**1**, 4.0 mmol·L^{-1}) and K_2PdCl_4 (4.0 mmol·L^{-1}) in D_2O (phosphate buffer 0.12 mol·L^{-1}, pD 7.6, 25 °C). Notation: Open circles refer to uncomplexed CMP, filled squares to the binary complex (CMP)Pd^{2+} complex and open squares to the mixed ligand complex (**1**)Pd(CMP).

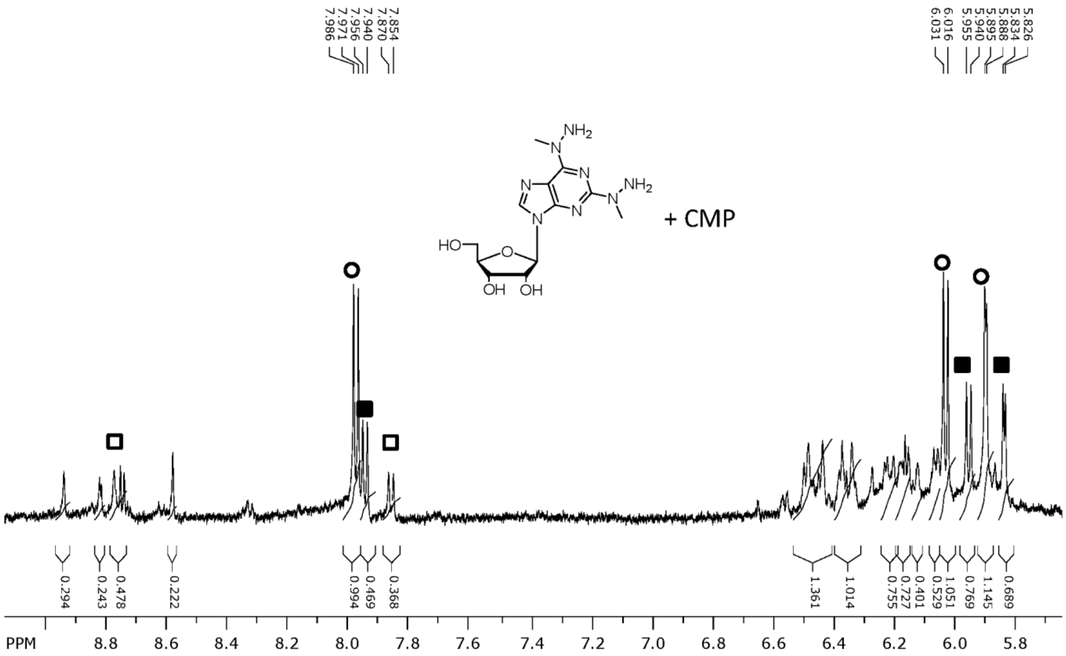

Figure S9. Aromatic and anomeric proton signals for the mixture of UMP (5 mmol·L^{-1}), 2,6-bis(1-methylhydrazinyl)-9-β-D-ribofuranosylpurine (**2**, 4.0 mmol·L^{-1}) and K_2PdCl_4 (4.0 mmol·L^{-1}) in D_2O (phosphate buffer 0.12 mol·L^{-1}, pD 7.6, 25 °C). Notation: Open circles refer to uncomplexed UMP and open squares to the mixed ligand complex (**2**)Pd(UMP).

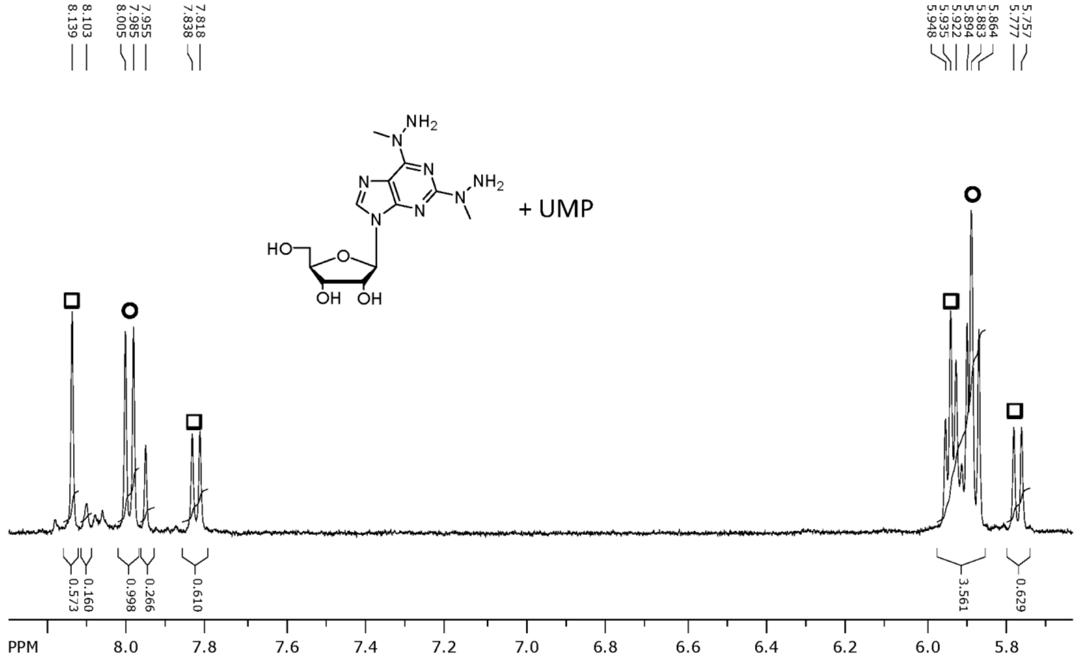

Figure S10. Aromatic and anomeric proton signals for the mixture of CMP (5 mmol·L^{-1}), 2,6-bis(1-methylhydrazinyl)-9-β-D-ribofuranosylpurine (**2**, 4.0 mmol·L^{-1}) and K_2PdCl_4 (4.0 mmol·L^{-1}) in D_2O (phosphate buffer 0.12 mol·L^{-1}, pD 7.6, 25 °C). Notation: Open circles refer to uncomplexed CMP and open squares to the mixed ligand complex (**2**)Pd(CMP).

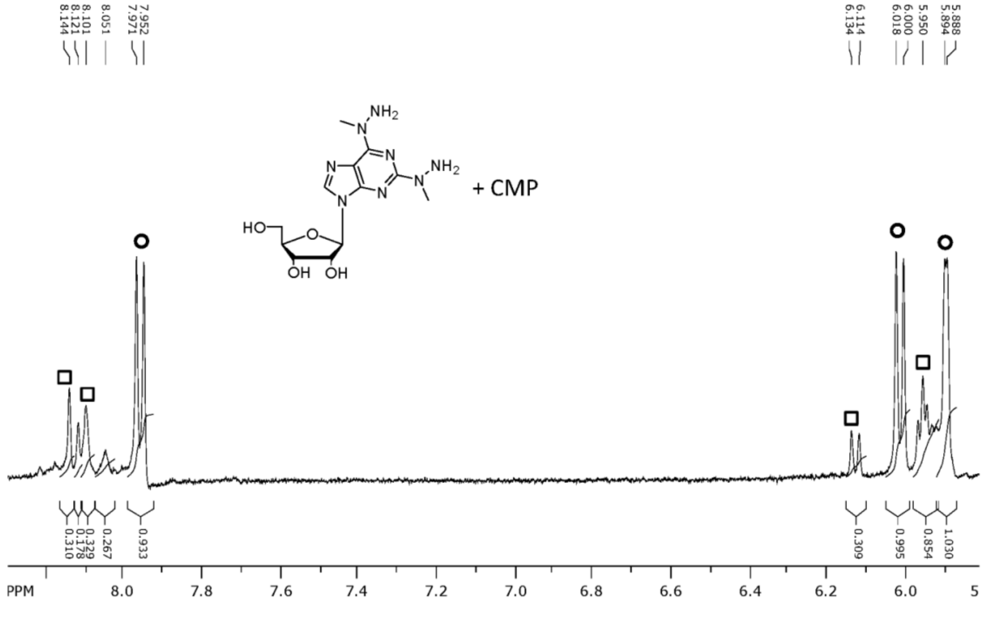

Figure S11. Aromatic and anomeric proton signals for the mixture of GMP (5 mmol·L^{-1}), 2,6-bis(1-methylhydrazinyl)-9-β-D-ribofuranosylpurine (**2**, 4.0 mmol·L^{-1}) and K_2PdCl_4 (4.0 mmol·L^{-1}) in D_2O (phosphate buffer 0.12 mol·L^{-1}, pD 7.6, 25 °C). Notation: Open circles refer to uncomplexed GMP and open squares to the mixed ligand complex (**2**)Pd(GMP).

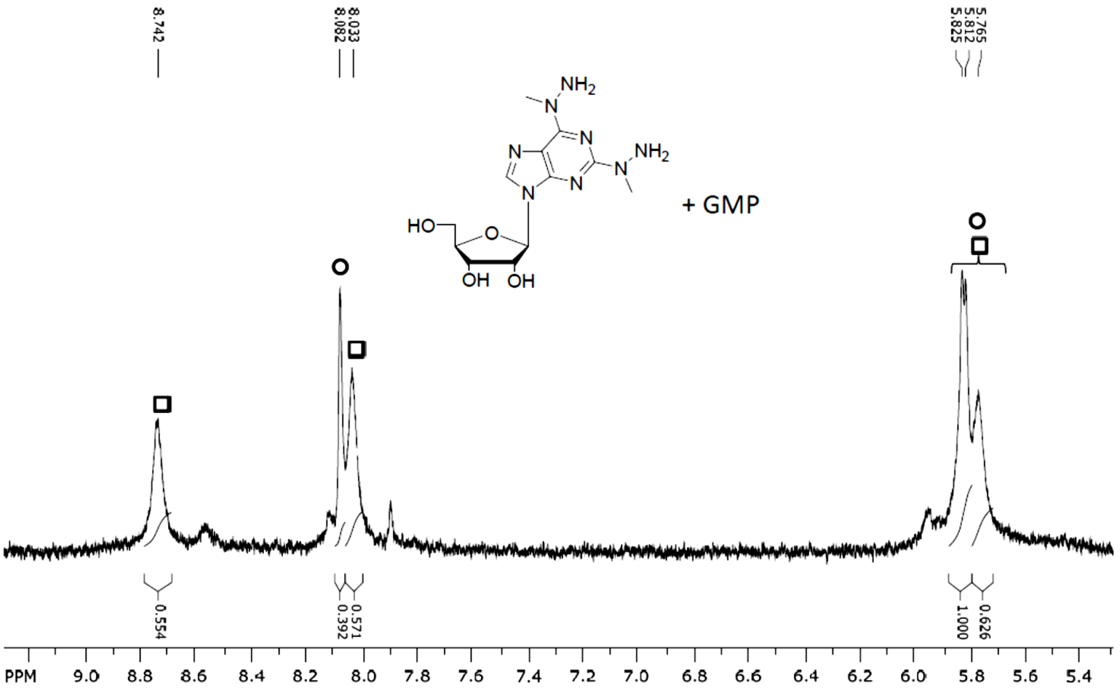

Supplement: Supplementary File 1 [file molecules-19-16976-s001.pdf]
